# The Effectiveness and Safety of Topical Capsaicin in Postherpetic Neuralgia: A Systematic Review and Meta-analysis

**DOI:** 10.3389/fphar.2016.00538

**Published:** 2017-01-10

**Authors:** Yi Lai Yong, Loh Teng-Hern Tan, Long Chiau Ming, Kok-Gan Chan, Learn-Han Lee, Bey-Hing Goh, Tahir Mehmood Khan

**Affiliations:** ^1^Novel Bacteria and Drug Discovery Research Group, School of Pharmacy, Monash University MalaysiaSelangor Darul Ehsan, Malaysia; ^2^Unit for Medication Outcomes Research and Education (UMORE), Pharmacy, School of Medicine, University of Tasmania (UTAS)Hobart, TAS, Australia; ^3^Division of Genetics and Molecular Biology, Institute of Biological Sciences, Faculty of Science, University of MalayaKuala Lumpur, Malaysia; ^4^Center of Health Outcomes Research and Therapeutic Safety (Cohorts), School of Pharmaceutical Sciences, University of PhayaoPhayao, Thailand

**Keywords:** pain, postherpetic neuralgia, topical agent, capsaicin, *Capsicum*

## Abstract

In particular, neuropathic pain is a major form of chronic pain. This type of pain results from dysfunction or lesions in the central and peripheral nervous system. Capsaicin has been traditionally utilized as a medicine to remedy pain. However, the effectiveness and safety of this practice is still elusive. Therefore, this systematic review aimed to investigate the effect of topical capsaicin as a pain-relieving agent that is frequently used in pain management. In brief, all the double-blinded, randomized placebo- or vehicle-controlled trials that were published in English addressing postherpetic neuralgia were included. Meta-analysis was performed using Revman^®^ version 5.3. Upon application of the inclusion and exclusion criteria, only six trials fulfilled all the criteria and were included in the review for qualitative analysis. The difference in mean percentage change in numeric pain rating scale score ranges from -31 to -4.3. This demonstrated high efficacy of topical capsaicin application and implies that capsaicin could result in pain reduction. Furthermore, meta-analysis was performed on five of the included studies. All the results of studies are in favor of the treatment using capsaicin. The incidence of side effects from using topical capsaicin is consistently higher in all included studies, but the significance of safety data cannot be quantified due to a lack of *p*-values in the original studies. Nevertheless, topical capsaicin is a promising treatment option for specific patient groups or certain neuropathic pain conditions such as postherpetic neuralgia.

## Introduction

Pain is described as an unpleasant sensory and emotional experience associated with actual or potential tissue damage (Bode and Dong, 2011; Desai et al., 2012; Simon, 2012). There are two common types of pain, acute pain and chronic pain. Basically, acute pain is crucial in alerting an individual to withdraw from a harmful situation while chronic pain could be constitute of serious, separate disease entity (Desai et al., 2012). For more detailed information, chronic pain is highly prevalent, affecting over 1.5 billion people worldwide (National Center for Health Statistics, 2007; Medeiros and Winsler, 2014). It is noteworthy that chronic pain affects more people than other chronic conditions such as diabetes, heart disease and cancer (Simon, 2012). The annual costs of chronic pain in the United States (including the total incremental cost of health care and cost of lost productive time) are estimated to be at least USD 560 billion (Medeiros and Winsler, 2014). As suggested by studies, chronic pain has a huge detrimental effect on the quality of life of patients (National Center for Health Statistics, 2007; Medeiros and Winsler, 2014). Undeniably, chronic pain is a significant healthcare issue, as it poses an enormous burden on patients, society and the healthcare system (Medeiros and Winsler, 2014).

In particular, neuropathic pain is a major form of chronic pain. Neuropathic pain results from dysfunction or lesions in the central and peripheral nervous system (Bridges et al., 2001; Campbell and Meyer, 2006; Treede et al., 2008; Nickel et al., 2012). Neuropathic pain conditions include HIV neuropathy (neurological complication of HIV) and postherpetic neuroglia (Derry et al., 2013). Postherpetic neuralgia (PHN) is a debilitating complication of herpes zoster ophthalmicus (HZO), commonly known as shingles, especially in elderly patients (Bucci et al., 1988). Besides, it is estimated that 10–15% of patients who have shingles will experience PHN (FDA, 2009). In particular, this disease is characterized by a distinctive syndrome—a painful skin rash mainly caused by reactivation of varicella zoster virus (VZV), especially if there is immunity to VZV drops due to aging or immunosuppression (Johnson and Whitton, 2004). Unfortunately, current treatment modalities such as tricyclic antidepressants and anticonvulsants are largely unsuccessful (due to adverse effects, poor tolerability and slow onset of action) (Bucci et al., 1988; Backonja et al., 2008). Unlike nociceptive pain, neuropathic pain such as PHN cannot be relieved by conventional analgesics such as paracetamol (Bridges et al., 2001). To make the scenario even worse, the prolonged and unresolved excruciating pain resulting from PHN often leads to depression, and in extreme cases suicide (Bucci et al., 1988).

Over the last three millennia, human civilization has relied on natural products derived from plants, animals and microbial origins to alleviate and cure sickness (Tan et al., 2015a,b; Tang et al., 2016; Yuan et al., 2016). From as far back as 60,000 years ago, in the Middle Paleolithic Age, there is evidence that humans were using plants as medicines (Fabricant and Farnsworth, 2001). The use of plants in traditional medical systems such as Ayurveda, Unani, Kampo and traditional Chinese medicine have flourished for 1000s of years (Fabricant and Farnsworth, 2001; Tan et al., 2016a,b). Although medical science views such systems as lacking credibility and scientific logic, it is notable that a lot of plant-originated drugs in current clinical medicine were derived from traditional medicine and serve as platforms for modern drug development. Most importantly, those products have become resources for developing new lead compounds and scaffolds (Fabricant and Farnsworth, 2001; Chan et al., 2016; Tan et al., 2016c). Given that over one-third of the world’s population, mainly in rural areas, lacks regular access to affordable modern medicines, the majority of people from countries in Africa, Asia and Latin America largely rely on traditional medicine, which is widely available to help meet some of their primary health care needs (Zhang, 2004; Verma and Singh, 2008).

Plants are reported to have been traditionally used as analgesics or resources for compounds with pain-relieving effects (Hamilton and Baskett, 2000; Tan et al., 2015a; Chan et al., 2016). For instance, opiate receptor agonists from poppy seeds and cyclooxygenase inhibitors from willow bark are widely used to alleviate pain in ancient medicine (Brownstein, 1993; Thun, 2000). Chili is also one of the sources for analgesic medications derived from plants. The ‘chili’ or ‘chili pepper’ plant, which is categorized under the genus Capsicum, belongs to a dicotyledonous group of flowering plants. The taxonomic position of Capsicum can be represented as follows: Kingdom – Plantae; Division – Magnoliophyta; Class – Magnoliopsida; Order – Solanales; Family – Solanaceae; Genus – *Capsicum*; Species – *chinense/annuum/pubescens/*etc. (Basu and Krishna, 2003).

Since ancient times, chili has been recognized for its broad range of therapeutic properties, and has been used for centuries to remedy pain. Several external and internal applications have been reported in various streams of traditional medicine (Khan et al., 2014; Maji and Banerji, 2016). Externally, it is used to treat different types of pain including rheumatism (joint pain), lumbago (lower back pain) and neuralgia (pain spread through nerves) (Khare, 2004). It can also be used as a local stimulant, counter-irritant, and rubefacient (Iwu, 1993; Panda, 1999). Internally, chili is used to treat dyspepsia, loss of appetite, flatulence, atherosclerosis, stroke, heart disease, and muscle tension (Panda, 1999; Khare, 2004). In Unani medicine, chili is utilized to prevent colds, sinus infections and sore throats, and to improve digestion and blood circulation (Khare, 2004). In folk medicine, it is suggested to treat cancer, asthma, bronchitis and cough. In addition, its regular consumption is also believed to be beneficial for anorexia, hemorrhoids, liver congestion, and varicose veins (Duke and DuCellier, 1993).

The broad traditional usage of chili has prompted the identification of capsaicin as the main active component of a variety of chili peppers such as habaneros and jalapeños. Capsaicin is responsible for causing the ‘hot’ and sharp pungent sensation (Bode and Dong, 2011). Those properties have been suggested to act through counter-irritation, which results in analgesic effects. Modern usage of capsaicin focuses on the treatment of various types of pain (Cortright et al., 2007). In fact, capsaicin has been studied clinically as a topical treatment for the pain of rheumatoid and osteoarthritis (Persson et al., 2016), psoriasis, diabetic neuropathy, and postherpetic neuralgia (Srinivasan, 2015). However, the efficacy of capsaicin in the treatment of these chronic pain disorders is still elusive.

In order to have a better understanding of the various action of capsaicin, studies have focused on the mechanism of capsaicin in pain induction (Cortright et al., 2007). It is known as a selective TRPV1 receptors ligand. The burning sensation is triggered upon the binding of capsaicin to the receptors. It is believed that the analgesic effect of capsaicin is due to its ability to cause reversible desensitization or defunctionalization, where the TRPV1-containing sensory axons become unresponsive to stimuli during the long-lasting refractory period (Bode and Dong, 2011; Derry et al., 2013). As a result, after repeated exposure to capsaicin, pain transmission is prevented and the pain response is reduced (Derry et al., 2013). As such, it is different from other naturally occurring irritant components. This feature of capsaicin has been exploited for therapeutic use for many years. Other than that, its ability to cause reversible nerve-fiber degeneration also contributes to the analgesic effect (Bode and Dong, 2011; Derry et al., 2013).

Conventionally, topical capsaicin has been used in pain management in numerous neuropathic pain conditions. FDA (2009), the Qutenza patch, a pure, synthetic capsaicin-containing prescription drug, was approved by the Food and Drug Administration (FDA) for long-term pain relief for PHN patients. In spite of that, its safety has been much debated. This is mainly because capsaicin is associated with some severe side effects such as capsaicin-induced dermal pain and contact dermatitis (human hand) (Bode and Dong, 2011). Topical capsaicin products such as Qutenza may cause a significant rise in blood pressure, necessitating the need for blood-pressure monitoring by health care professionals (FDA, 2009). Emerging evidence has also suggested that long-term application of topical capsaicin may be harmful. As mentioned previously, capsaicin exerts its therapeutic action by the desensitization process. Therefore, prolonged use of topical capsaicin may lead to persistent desensitization. Furthermore, multiple epidemiology studies have suggested that capsaicin may have carcinogenic properties. Its effectiveness has also not been fully established (Bode and Dong, 2011). Additionally, the effectiveness of topical capsaicin varies among patients with different conditions. It also seems to have inconsistent effectiveness across neuropathic pain conditions.

At the same time, there is a lack of comprehensive reviews on the effectiveness of capsaicin on PHN. Furthermore, earlier reviews have not included recent evidence (from research conducted using more rigorous and stringent standards). There is a need to conduct a literature review that includes the recent studies. By doing so, we will be able to investigate the effectiveness and safety of topical capsaicin using recent evidence.

**FIGURE 1:**
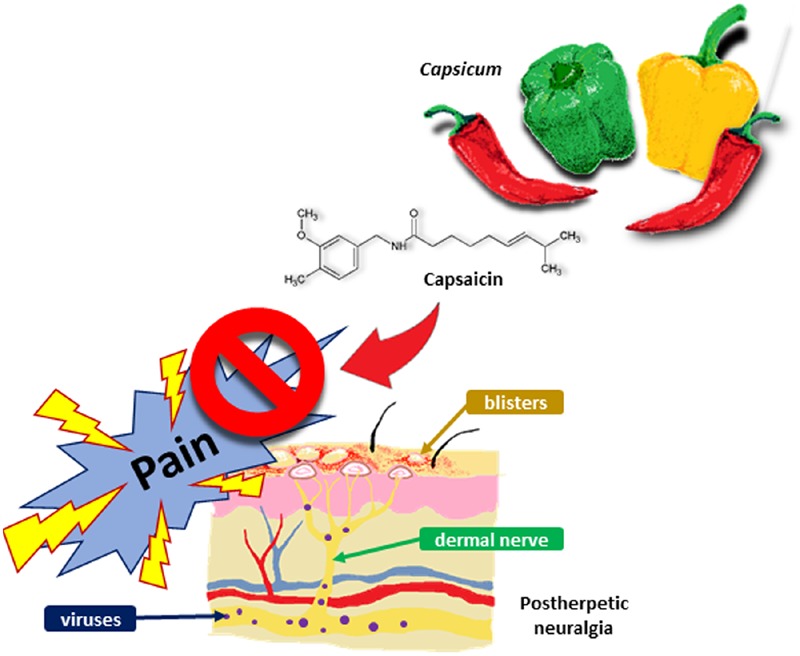
**The potential of capsaicin as first-line treatment in PHN**.

The PICO framework is utilized to develop the main question (Gray, 2003). By referring to the relevant review articles and papers, the research question is further refined. The research question is: ‘Is topical capsaicin efficacious and safe (compared to a placebo) to be used as a first-line treatment in the management of chronic neuropathic pain (particularly PHN) in adult and elderly patients?’ By critically appraising the included studies, we will investigate the efficacy and safety of topical capsaicin in pain management. Topical capsaicin is usually prescribed as a third-line treatment or adjunctive treatment (Argoff, 2011). Based on the findings, we will evaluate its risk-benefit ratio and explore the feasibility of using topical capsaicin as a first-line treatment in PHN (**Figure 1**). It could potentially be used as a first-line option if it shows adequate efficacy and safety.

## Method

A systematic literature search was performed using databases including PubMed, Medline, Embase, Science Direct and Google Scholar. Cochrane Library and Wiley Library were also used to retrieve related papers.

### Data Sources

A search string was developed using the keywords in the topic, their synonyms and different registered brand names of capsaicin. The search string used was (topical capsaicin OR topical capsicin^∗^ OR topical capsicum OR topical analgesic^∗^ OR Capsagel OR Salonpas-Hot OR Zostrix OR Trixaicin OR Qutenza) AND (continu^∗^ OR last^∗^ OR prolonged OR chronic OR persist^∗^) AND (postherpetic neuralgia OR PHN) AND (pain OR ache). Search techniques such as Boolean operators, truncation, citation tracking, and chaining were also applied to retrieve relevant resources.

### Study Selection

Eligible studies were selected based on a set of inclusion criteria. Based on the inclusion criteria, studies were included only if they were double-blinded, randomized placebo- or vehicle- controlled trials that were published in English. The type of chronic pain considered was neuropathic pain (limited to only postherpetic neuralgia) and the considered dosage forms were creams and patches (topical). Both high- and low-concentration capsaicin were considered in the review. The minimum duration of the included studies was 6 weeks while the minimum age of participants was 18 (adults and elderly). Only studies that used a placebo or vehicle as the control arm were considered.

A PRISMA diagram was used to depict the flow of information through different phases for the systematic review (Liberati et al., 2009).

### Analysis Strategy

Finally, the methodological quality of the included studies was assessed using the Jadad scale. The studies were allocated a score (from zero to five) that indicated the quality of the study (based on randomization, blinding and withdrawal or dropout in the study).

The risk of bias of the studies was assessed using Cochrane Collaboration’s tool, where seven domains of bias are addressed. Each domain was assigned high, low or unclear risk. The numerical data for primary and secondary outcomes was extracted and tabulated, and the missing data was calculated where possible. For example, the confidence interval was calculated using standard error and standard deviation. The intention to treat (ITT) principle was applied in the analysis of data.

Meta-analysis was performed on five studies that had sufficient data. A forest plot was constructed to graphically summarize the results of the included studies for the primary outcome (Schriger et al., 2010). The heterogeneity of the studies was also assessed. Using RevMan, the weighted average of studies was calculated. Additionally, a risk of bias graph and summary was constructed.

### Outcomes

The primary outcome is a clinically significant reduction in pain and the response to treatment. This is indicated by:

(i) Difference in mean percentage change in 11-point numeric pain rating scale (NPRS) or visual analog pain scale (VAS) from baseline to weeks 2–12 or baseline to weeks 2–6;
(ii) Reduction in NPRS score of more than 30 and/or 50% at the end of the trial;
(iii) Mean reduction in seven-point patient global impression of change (PGIC).

A secondary outcome is any side effect or adverse effect, including musculoskeletal disorder, hyperalgesia, fatigue, vomiting, transient hypertension, stinging, and erythema at application site.

## Results

Initially, a total of 109 records were identified using the search string (105 were found from Pubmed and Cochrane databases). There were 41 results remaining after duplicate results were removed. After screening the results using the inclusion and exclusion criteria described earlier, 34 results were excluded. The eliminated results include six reviews, two integrated studies, two short articles, one case series, one letter to an editor, four studies published only as abstract, one study that directly compared the efficacy of two agents, one preliminary study, two open label studies and a number of randomized controlled trials that were out of the research scope. Watson et al. (1993), which consists of both the double-blind phase and the long-term open-label phase, was included. However, only the results from the double-blind phase were analyzed. By further analyzing the full text of the seven remaining papers, only six fulfilled all the inclusion and exclusion criteria and were included in the review for qualitative analysis. Backonja et al. (2010), whose study lasted only 4 weeks, was excluded. All studies were included in quantitative analysis except Bernstein et al. (1989), due to the limited quantitative data.

**FIGURE 2:**
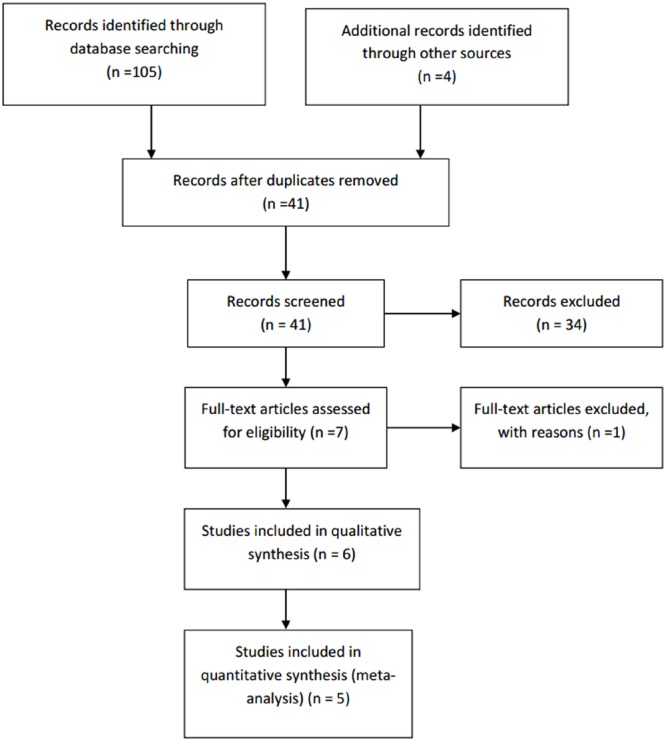
**PRISMA flow chart**.

Due to the limited number of recent studies, publications dated from 1989 to 2010 were included. However, most of the included studies are recent studies. The process of the literature search is depicted in the PRISMA flow chart attached (**Figure 2**).

### Characteristics of Participants

A total number of six studies (1449 patients) that fulfilled the inclusion criteria and exclusion criteria were included. Only adult patients aged 18 and above were considered. Elderly patients aged over 75 were also included in some studies (e.g., Bernstein et al., 1989). The gender ratio and baseline characteristics were unbalanced in some studies. The participants experienced chronic neuropathic pain for at least 3 months. The number of patients in the treatment arm ranged from 16 to 222 (**Table 1**).

**Table 1:** Characteristics of studies.

Studies	Type of studies	Mean age	Type of neuropathy conditions	Male/female	Participants (*N*)	Capsaicin concentration in treatment arm	Treatment duration	Jadad score
Backonja et al., 2008	Double-blind RCT	71	PHN	190/212	404 (*T* = 206, *C* = 196)	8% (high)	12 weeks	4
Webster et al., 2010a	Double-blind RCT	71	PHN	150/149	299 (*T* = 222, *C* = 77)	8% (high)	12 weeks	4
Webster et al., 2010b	Double-blind RCT	70	PHN	72/83	155 (*T* = 102, *C* = 53)	8% (high)	12 weeks	4
Irving et al., 2011	Double-blind RCT	70	PHN	190/226	416 (*T* = 212, *C* = 204)	8% (high)	12 weeks	4
Watson et al., 1993	Double-blind RCT	71	PHN	53/90	143 (*T* = 74, *C* = 69)	0.075% (low)	6 weeks	4
Bernstein et al., 1989	Double-blind RCT	72	PHN	12/20	32 (*T* = 16, *C* = 16)	0.075% (low)	6 weeks	4

*RCT, randomized controlled trials; PHN, postherpetic neuralgia; T, treatment; C, control.*

### Qualitative Analysis

Based on **Table 2**, all the results of studies favor the treatment (capsaicin). The difference in mean percentage change in NPRS score ranges from -31 to -4.3. Topical capsaicin demonstrated high efficacies in Bernstein et al. (1989) (highest mean reduction in NPRS score of -31), and lowest efficacy in Irving et al. (2011) (mean reduction in NPRS score of -4.3). The results imply that capsaicin could result in pain reduction.

**Table 2:** Comparison of primary end points 1 and 2.

Studies	Difference in mean percentage change in NPRS score or visual analog pain scale			Number and percentage of participants who have a reduction in NPRS score of more than 30% at the end of trial, and the risk ratio			Number and percentage of participants who have reduction in NPRS score of more than 50% at the end of trial, and the risk ratio		
	Treatment	Control	Difference	Treatment	Control	Risk ratio	Treatment	Control	Risk ratio
Backonja et al., 2008	-29.9 (-34.00 to -25.79)	-20.4 (-24.61 to -16.19)	-9.5 (-15.47 to -3.61)	91 (44%)	69 (33%)	1.51 (1.00 to 2.27)	N/A	N/A	N/A
Webster et al., 2010a	-25.0 (-29.02 to -20.98)	-14.7 ± 3.51	-10.3	83 (37%)	22 (29%)	0.92	55 (25%)	8 (10%)	2.84
Webster et al., 2010b	-36.6 (-44.02 to -29.19)	-32.3 (-42.63 to -22.05)	-4.3	50 (49%)	26 (49%)	1.00	40 (39%)	19 (36%)	5.96
Irving et al., 2011	-32.3 (-36.49 to -28.12)	-25.0 (-29.29 to -20.76)	-7.3	100 (47%)	72 (35%)	1.64	64 (30%)	43 (21%)	1.62
Watson et al., 1993	-15.0	-5.2	-9.8	N/A	N/A	N/A	N/A	N/A	N/A
Bernstein et al., 1989	-30	1	-31	N/A	N/A	N/A	N/A	N/A	N/A

*% (95% confidence interval), *p*-value; %, *p*-value.*

*There were two different sets of data in Watson et al. (1993)—patients who have PHN for longer than 12 months, and patients who have PHN for longer than 6 months. The data shown was obtained from patients who have PHN for longer than 12 months.*

There is discrepancy in the key findings (see **Table 3**). While most studies report that capsaicin is efficacious and/or safe, Webster et al. (2010b) suggest otherwise. However, it should be noted that the results of Webster et al. (2010b) are statistically insignificant as the *p*-value is greater than 0.05. As shown in **Table 2**, a risk ratio of more than one is calculated for almost all the studies except Webster et al. (2010a). The number of participants who have a reduction in NPRS score of more than 30% was not measured in either Bernstein et al. (1989) or Watson et al. (1993). Webster et al. (2010b) has a *p*-value of greater than 0.05, indicating that the results may be insignificant. The number of participants who have a reduction in NPRS score of more than 50% was only measured in Webster et al. (2010a,b) and Irving et al. (2011). Similarly, the results of Webster et al. (2010b) may be insignificant (*p*-value > 0.05).

**Table 3:** Summary of outcomes and key findings.

Studies	Summary of primary and secondary outcomes at the end of studies	Key findings
Backonja et al., 2008	**NPRS:** Mean reduction of score is higher in treatment group (29.9%) than control group (20.4%).	Capsaicin (NGX-4010) is safe and efficacious in reducing pain in postherpetic neuralgia patients.
	**PGIC**: Slightly improved, much improved and very much improved higher in treatment group (55%) compared to control (43%).
	**Adverse reaction:** Short lasting pain and erythema is generally mild and moderate in treatment group.	
Webster et al., 2010a	**NPRS:** Mean reduction of score is significantly higher in treatment group (25.0%) than control group (14.7%) **PGIC**: Total improved (slightly, much and very much improved) higher in treatment group (55%) than control group (41%). **CGIC:** Total improved (slightly, much and very much improved) higher in treatment group (52%) than control group (42%). **Adverse reaction:** Mild to moderate, transient side effects are observed. Generally well-tolerated. 1% of participants withdrawn due to side effects. 59% of treatment group patient reported adverse events. No serious adverse events are related to treatment.	Capsaicin (NGX-4010) is efficacious in pain reduction in postherpetic neuralgia. Lowest effective dose is required in 60-min treatment.
Webster et al., 2010b	**NPRS**: Mean reduction of score is higher in treatment group (36.6%) than control group (32.3%) *(but no statistical significance).* **PGIC**: Much improved and very much improved in treatment group (43%) is higher than control group (30%) *(but no statistical significance)*. **CGIC:** Total improved (slightly, much and very much improved) higher in treatment group (43%) than control group (24%). **Adverse reaction:** Generally well-tolerated in most participants and side effects are manageable in most cases. 4% of participants withdrawn due to burning and pain at application site. 75% of treatment group patient reported adverse events. No serious adverse events related to treatment.	Although, capsaicin (NGX-4010) appeared to be safe and well-tolerated, it failed to show significant efficacy in participants with postherpetic neuralgia for less than 6 months.
Irving et al., 2011	**NPRS:** Mean reduction of score is higher in treatment group (32.3%) than control group (25%). **PGIC**: Total improved (slightly, much improved and very much improved) higher in treatment group (61%) than control group (47%). **CGIC**: Total improved (slightly, much improved and very much improved) higher in treatment group (63%) than control group (48%). **Adverse reaction:** Higher occurrence in treatment group. Generally well-tolerated. 2% of participants withdrawn due to side effects. There is mild to moderate skin reactions at application site. No serious adverse events are caused by intervention.	Capsaicin (NGX-4010) is efficacious in pain reduction for postherpetic neuralgia.
Watson et al., 1993	**Patients with PHN for more than 6 months** **Visual analog pain scale:** Mean reduction is slightly higher in treatment group (20.9%) than control group (5.8%). **PGIC:** Total improved higher in treatment group than control group. 65% of treatment group versus 34% of the patients experienced reduction in PHN pain. **CGIC:** Total improved higher in treatment group than control group. 38% of treatment group versus 20% of the patients experienced reduction in PHN pain. **Adverse reaction:** There were no serious adverse effects observed or reported during trial. Only burning, stinging and erythema at application sites was directly attributable to capsaicin cream. **Patients with PHN for more than 12 months** **Visual analog pain scale:** Mean reduction is slightly higher in treatment group (15%) than control group (5.2%). **PGIC:** Total improved higher in treatment group than control group. 39% of treatment group versus 6% of the patients experienced reduction in PHN pain. **CGIC:** Total improved higher in treatment group than control group. 64% of treatment group versus 25% of the patients experienced reduction in PHN pain.	Capsaicin cream is a safe and effective treatment for the pain of PHN and should be considered for initial management of patients with this condition.
	**Adverse reaction:** There were no serious adverse effects observed or reported during trial. Only burning, stinging and erythema at application sites was directly attributable to capsaicin cream.	
Bernstein et al., 1989	**Visual analog pain scale for pain measurement:** Mean reduction is higher in treatment group (30%) than control group (1% increase). **Visual analog pain scale for pain relief:** Mean reduction is higher in treatment group (54%) than control group (6%). **PGIC**: Total improved (much and very much improved) higher in treatment group (46%) than control group (6%). **CGIC:** Total improved higher in treatment group (77%) than control group (31%). **Adverse reaction:** There are mild to moderate skin reactions at application site. No systemic adverse events are caused by intervention.	Capsaicin could be used for initial treatment of postherpetic neuralgia due to low systemic toxicity and no drug interactions.

In terms of PGIC (**Table 4**), the number of patients who reported improvements (slightly, much or very much) in pain reduction is higher in the treatment group in all the studies. This suggests that capsaicin may be effective in pain reduction (as perceived by the patients). All results are significant (*p* < 0.05). The *p*-value for Webster et al. (2010a) is unavailable, so the significance of its results could not be determined. In terms of the secondary end points (**Table 4**), the number and percentage of patients who experience side effects is higher in the treatment group. The trend is consistent in all studies. This suggests that the use of topical capsaicin may be unsafe due to its side effects (as suggested by earlier studies) (Bode and Dong, 2011). The *p*-values (and hence significance) of the safety data from all seven studies are unknown.

**Table 4:** Comparison of primary end point 3 and secondary endpoint: mean reduction in seven-point patient global impression of change (PGIC).

Studies	Number and percentage of patients who have improved (slightly, much and very much) at the end of the study		Number and percentage of participants who experienced any adverse events	
	Treatment	Control	Treatment	Control
Backonja et al., 2008	114 (55%)	85 (43%),	203 (99%)	174 (88%)
Webster et al., 2010a	122 (55%)	32 (41%)	131 (59%)	43 (56%)
Webster et al., 2010b	41 (43%)	15 (30%)	76 (75%)	28 (53%)
Irving et al., 2011	123 (61%)	91 (47%)	208 (98%)	177 (87%)
Watson et al., 1993	91 (64%)	17 (25%)	45 (61%)	23 (33%)
Bernstein et al., 1989	4 (46%)	1 (6%)	NA	NA

**FIGURE 3:**
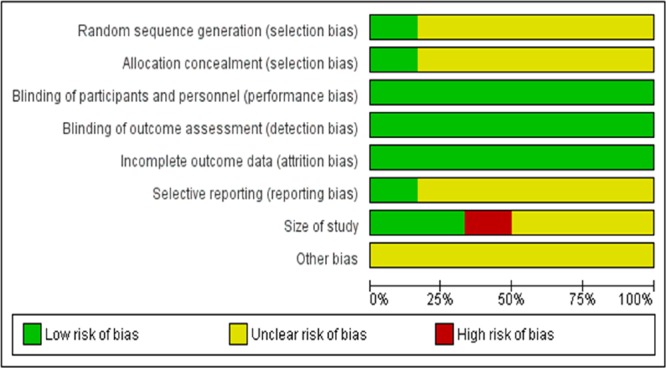
**Overall Risk of bias graph**.

Based on **Figure 3**, most of the risks of bias of the included studies are acceptably low. All studies have low risks of performance bias, attrition bias and detection bias. However, all studies have an unclear risk of selection bias (random sequence generation) and reporting bias. Most studies (83.33%) have unclear risks of selection bias (allocation concealment) and reporting bias. Only 16.67% of the studies have low risk of these two biases. In terms of size of study, only 33.33% of the studies have a low risk of bias. The remaining studies have unclear risk (50%) or high risk (16.67%). All studies have an unclear risk of other bias.

**FIGURE 4:**
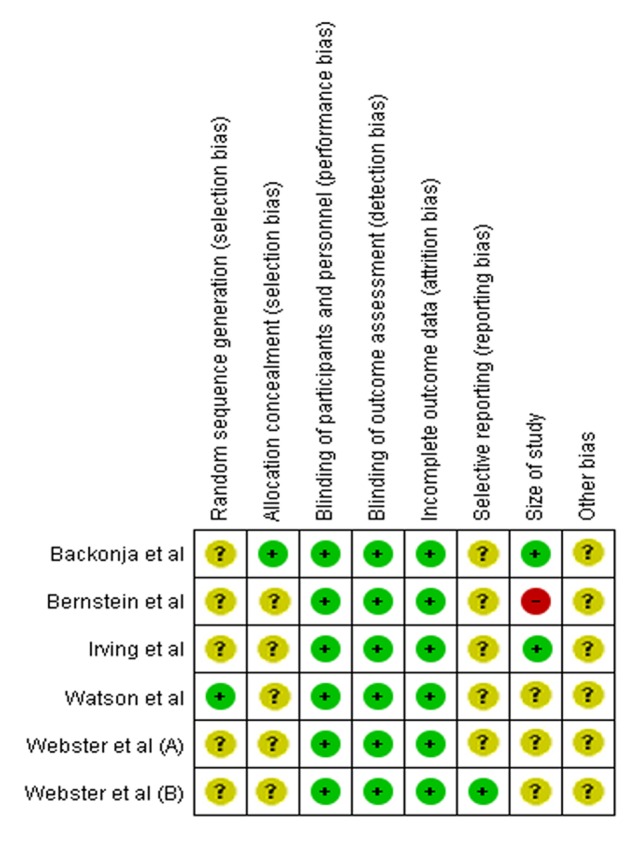
**Summary of Risk of Bias for individual studies**.

In **Figure 4**, Backonja et al. (2008) has the lowest risk of bias (low risk in five items and unclear risk of bias in three items). On the other hand, Bernstein et al. (1989) (low risk in three items, unclear risk in four items, high risk in one item) has the highest risk of bias. The rest of the studies have acceptably low risks of bias. Based on these analyses, most studies have moderately high validity. Overall, the methodological quality of the included studies is satisfactory, as all studies have a score of 4 out of 5. The heterogeneity is found to be 0%, indicating that it is likely to be insignificant (Higgins and Green, 2011).

### Quantitative Analysis

**FIGURE 5:**
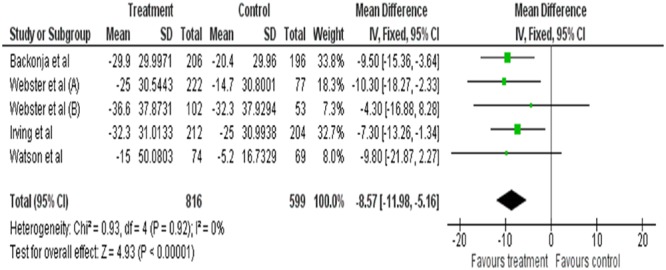
**Forest plot of treatment (capsaicin) vs. control**.

Meta-analysis was performed on five of the included studies. As seen in the forest plot, topical capsaicin displays varying degree of efficacies in each study. All the results of studies favor the treatment (capsaicin). Overall the efficacy of topical capsaicin is moderately high. Based on **Figure 5**, topical capsaicin demonstrated its highest efficacy in Webster et al. (2010a) (highest mean reduction in NPRS score of -10.30), and its lowest efficacy in Webster et al. (2010b) (mean reduction in NPRS score of -4.3). It is noteworthy that the results of Watson et al. (1993) and Webster et al. (2010b) are not statistically significant. All six studies have acceptably low risks of bias (as discussed previously), so the results are likely to be valid.

It must be noted that further statistical analysis on the safety on capsaicin could not be performed due to a lack of relevant data. Therefore the statistical significance of the results could not be confirmed.

## Discussion

Management of neuropathic pain such as PHN is more challenging than other types of pain (Park and Moon, 2010). In general, patients with neuropathic pain have higher pain scores than patients with non-neuropathic pain (Park and Moon, 2010). In addition, patients with neuropathic pain are reported to have a lower quality-adjusted life-year (QALY) and a higher risk of depression (Bucci et al., 1988; Park and Moon, 2010). They also experience less pain relief with the standard treatment, so they usually need multiple drugs and adjunctive treatments for adequate pain management (Smith et al., 2007; Park and Moon, 2010).

For PHN patients, good adherence to medicine is particularly important for adequate pain management. Patients need to continuously reapply capsaicin cream throughout the day due to the low concentration of the active ingredient (0.025–0.075%) (Das et al., 2013). The four-times-daily application may threaten medication compliance (Jorge et al., 2011).

While capsaicin cream (which requires frequent application) may reduce adherence, a capsaicin patch could potentially improve a patient’s adherence, as such a patch only needs to be reapplied every 3 months (Das et al., 2013). This could be accounted for by the down-regulation of TRPV-1 receptors (Jorge et al., 2011). The use of a capsaicin patch allows the medication regimen to be simplified and hence improves medicine adherence. The use of topical capsaicin in place of tricyclic antidepressants and anticonvulsants (which are commonly used to treat PHN) also eliminates the need to titrate doses, thus minimizing the risk of side effects or withdrawal symptoms.

Besides, topical capsaicin does not have systemic effects (e.g., CNS effects). As reported in one study, CNS impairment is the least acceptable side effect of pain-relieving medicines among chronic pain patients (Jorge et al., 2011). Hence, topical capsaicin would be better received than other conventional treatment options (which have significant systemic effects). The increased patient acceptance of medicine could significantly improve adherence and eventually lead to better pain management.

As topical capsaicin is able to provide efficient pain relief with fewer central nervous system effects and a minimal drug regimen burden, it seems to be an ideal candidate as a first-line agent in the management of PHN (Bernstein et al., 1989; Jorge et al., 2011). However, in practice, topical capsaicin is not commonly used as first-line treatment in chronic pain management (NICE, 2013). In fact, it is not advisable to use topical capsaicin (capsaicin patch) for initial treatment. For the initial treatment of PHN, the recommended first- and second-line agents are gabapentin, a lidocaine patch, opioid analgesics, and tricyclic antidepressants (Dworkin et al., 2003). Like the cream formulation, capsaicin patches may cause a burning sensation (Das et al., 2013). As placement of the patch can be quite painful, a local topical anesthetic or opioid pain relievers must be used concurrently during application (FDA, 2009). A capsaicin patch may also increase blood pressure during initial application (Das et al., 2013). In fact, the FDA recommends blood-pressure monitoring for at least an hour after the application of a capsaicin patch. In general, the role of topical preparations in patient adherence remains unclear, as there is a lack of compliance studies that compare traditional routes (e.g., oral) and topical treatment in chronic pain management (Jorge et al., 2011).

### Evidence from the Included Studies

Based on the difference in mean NPRS score, most studies have demonstrated that topical capsaicin has a moderately high efficacy in pain reduction. This indicates that topical capsaicin alone may adequately reduce pain. In terms of PGIC, the number of patients who reported improvements in pain reduction is higher in the treatment group in all seven studies. This also suggests that topical capsaicin is able to adequately control pain. However, the significance of these results could not be determined. Although, the results obtained for the two primary efficacy endpoints (NPRS and PGIC scores) correlate well with one another (i.e., both suggest adequate pain reduction), it is unknown whether they are equivalent in terms of accuracy and sensitivity. Moreover, the efficacy of topical capsaicin compared to other agents is unknown, as all the included studies only used a placebo in the comparator arm. However, a study suggests that topical capsaicin has significantly higher efficacy compared to oral products (Armstrong et al., 2011). The same study also found that the cost effectiveness of topical capsaicin is similar or acceptable compared to other existing therapies (Armstrong et al., 2011).

More patients in the treatment group experienced side effects than in the control group. However, the statistical significance of these results could not be confirmed because statistical analysis could not be performed on the secondary outcome, due to a lack of data. It is also unknown whether or not these side effects are well-tolerated, as this is not described in the studies. The severity of the side effects is also unclear. The safety profile of topical capsaicin remains unknown and its safety could not be established based on the limited evidence.

### Potential Use of Topical Capsaicin

Based on the available evidence, it is likely that the risks of using topical capsaicin as a first-line treatment outweigh its benefits, due to safety concerns. In other words, the use of topical capsaicin could possibly have an unfavorable risk-benefit ratio. Furthermore, there is insufficient evidence on the efficacy of topical capsaicin to support its use. Nevertheless, topical capsaicin could be a first-line treatment option for patients who are intolerant to oral treatment and systemic side effects, or who have poor compliance (Das et al., 2013). Besides, it may be suitable for patients with oral neuropathic pain. Topical preparations can potentially benefit pediatric patients (whose chronic pain management is no less challenging than adults), since a significant number of the pediatric population is unable to swallow tablets (Jorge et al., 2011; Zajicek et al., 2013). Moreover, PHN is one of the main causes of morbidity among the elderly (who are more resistant to treatment). Topical capsaicin could be used for treatment of postherpetic neuralgia in the elderly due to its low systemic toxicity and minimal drug interactions. It is also more tolerable than other agents.

In essence, topical capsaicin could be a potential first-line treatment of chronic pain in specific patient groups or patients with specific conditions, for, example, elderly PHN patients. Undeniably, the use of topical capsaicin in chronic pain management is very limited and it is unlikely that topical capsaicin would be widely used as a first-line treatment due to the paucity of evidence on its efficacy and safety profile. For the time being, it could be used as an adjunct to other conventional first-line treatment options, as a combination treatment will usually have a higher efficacy and tolerability (NICE, 2013).

## Limitations of Evidence

It should be noted that some of the studies, including Backonja et al. (2008) and Webster et al. (2010a,b), are funded or sponsored. This could be a potential source of bias, as inappropriate influence of funders is often regarded as a risk of bias (Higgins and Green, 2011; Lexchin, 2012). There may be potential conflicts of interest.

Blinding of outcome assessors can be especially important for assessment of subjective outcomes, such as degree of pain. All studies are adequately described as double-blind, but maintenance of blinding is not well-described in some studies (e.g., Bernstein et al., 1989). Besides, blinding may have been broken if the participants correctly guessed which group they were in. In that case, the participants may not be truly blinded. Generally, blinding is considered to be broken if more than 50% of guesses are correct. To ensure adequate blinding, low-concentration capsaicin is used in the control group instead of an inert placebo in studies such as Webster et al. (2010a,b) and Irving et al. (2011). However, this may confound the results of the studies. For example, in Webster et al. (2010b), spontaneous resolution of postherpetic neuralgia may have resulted in better reduction of pain in the control group. Additionally, a data review has shown that there may be a difference in pain score reporting (PGIC) between genders. This could be a potential source of bias. Hence, a gender-stratified analysis is required. In studies where an NPRS score was not assessed (Bernstein et al., 1989; Watson et al., 1993), a VAS score was instead used as a primary outcome.

## Conclusion

Capsaicin, the main component in chili peppers, has immense ethnopharmacological potential, and has served as one of the main adjunctive treatments for neuropathic pain such as PHN. The current review aimed to compile and investigate the efficacy and safety of topical capsaicin in management of chronic pain caused by PHN. Based on a literature search, all the six included studies suggest that topical capsaicin is efficacious but at the same time is associated with a higher incidence of side effects. This prompted the need for a meta-analysis study. Based on the analysis, five of the included studies indicated the treatment with capsaicin has better efficacy compared to a vehicle-controlled placebo. However, the results of two studies involving 298 out of the 1415 total pooled population are not statistically significant. Unfortunately, the answer to the research question remains inconclusive. Therefore, it is still unclear whether or not topical capsaicin should be used as a first-line treatment. Further evidence is required to determine the risk-benefit ratio and support the use of capsaicin as a first-line treatment.

## Author Contributions

YY, LT, LM contributed to the literature database search, data collection, data extraction, data analysis, and writing of the manuscript. TK, K-GC, B-HG, and L-HL performed data analysis and rationalization of the results. The topic was conceptualized by B-HG, L-HL, and TK.

## Conflict of Interest Statement

The authors declare that the research was conducted in the absence of any commercial or financial relationships that could be construed as a potential conflict of interest.
